# Metagenome-Assembled Genomes (MAGs): Advances, Challenges, and Ecological Insights

**DOI:** 10.3390/microorganisms13050985

**Published:** 2025-04-25

**Authors:** Salvador Mirete, Mercedes Sánchez-Costa, Jorge Díaz-Rullo, Carolina González de Figueras, Pablo Martínez-Rodríguez, José Eduardo González-Pastor

**Affiliations:** 1 Centro de Astrobiología (CAB), CSIC-INTA, Carretera de Ajalvir km 4, Torrejón de Ardoz, 28850 Madrid, Spain; mersanchez@cab.inta-csic.es (M.S.-C.); jdiaz@cab.inta-csic.es (J.D.-R.); gonzalezfc@cab.inta-csic.es (C.G.d.F.); pmartinez@cab.inta-csic.es (P.M.-R.); 2 University of Alcalá, Polytechnic School, Ctra. Madrid-Barcelona, Km. 33.600, Alcalá de Henares, 28871 Madrid, Spain

**Keywords:** metagenome-assembled genomes (MAGs), genome-resolved metagenomics, microbial ecology, biogeochemical cycles, hybrid sequencing technologies, taxonomic classification, microbial metabolic pathways, environmental sustainability, assembly and binning algorithms, quality assessment of MAGs

## Abstract

Metagenome-assembled genomes (MAGs) have revolutionized microbial ecology by enabling the genome-resolved study of uncultured microorganisms directly from environmental samples. By leveraging high-throughput sequencing, advanced assembly algorithms, and genome binning techniques, researchers can reconstruct microbial genomes without the need for cultivation. These methodological advances have expanded the known microbial diversity, revealing novel taxa and metabolic pathways involved in key biogeochemical cycles, including carbon, nitrogen, and sulfur transformations. MAG-based studies have identified microbial lineages form Archaea and Bacteria responsible for methane oxidation, carbon sequestration in marine sediments, ammonia oxidation, and sulfur metabolism, highlighting their critical roles in ecosystem stability. From a sustainability perspective, MAGs provide essential insights for climate change mitigation, sustainable agriculture, and bioremediation. The ability to characterize microbial communities in diverse environments, including soil, aquatic ecosystems, and extreme habitats, enhances biodiversity conservation and supports the development of microbial-based environmental management strategies. Despite these advancements, challenges such as assembly biases, incomplete metabolic reconstructions, and taxonomic uncertainties persist. Continued improvements in sequencing technologies, hybrid assembly approaches, and multi-omics integration will further refine MAG-based analyses. As methodologies advance, MAGs will remain a cornerstone for understanding microbial contributions to global biogeochemical processes and developing sustainable interventions for environmental resilience.

## 1. Introduction

Microbial ecology is a discipline that investigates the interactions and functions of microorganisms within their natural environments. Traditionally, this field has relied on microbiological methods conducted in laboratory settings, requiring the cultivation of microorganisms for their study. However, a significant limitation arises from the fact that the vast majority of microorganisms present in natural environments—more than 90%—cannot be readily cultured under standard laboratory conditions [1,2,3,4,5]. As a result, traditional techniques constrain our ability to study microbial communities comprehensively, thereby restricting our understanding of biological diversity and microbial functions.

To overcome these limitations and enable a more in-depth exploration of microbial communities, several culture-independent methodologies have been developed. These approaches, collectively referred to as metagenomic techniques, are based on the analysis of the DNA directly isolated from environmental samples [6]. Among these, functional metagenomics involves the study of gene functions through the cloning of environmental DNA into laboratory-adapted microorganisms such as *Escherichia coli* [7]. Alternatively, sequencing-based metagenomics circumvent the need for cloning by leveraging high-throughput sequencing technologies to access the genetic information contained in environmental DNA without requiring microbial cultivation.

Advances in sequencing technologies and bioinformatics have significantly enhanced the power of these metagenomic approaches, enabling the assembly of complete microbial genomes and a better understanding of microbial diversity, providing key insights into their metabolic functions. The genomes reconstructed from metagenomic data are known as metagenome-assembled genomes (MAGs). The study of MAGs has been essential for identifying key metabolic processes involved in biogeochemical cycles, such as those of sulfur, carbon, and nitrogen, and has facilitated the discovery of novel microbial taxa and their ecological roles in diverse environments, including agricultural soils, engineered environments, thermal springs, and the human gut [8].

Importantly, the study of MAGs contributes directly to sustainability research by uncovering microbial processes that drive ecosystem resilience, carbon and nitrogen cycling, and bioremediation. It is well-established that microbial communities play fundamental roles in maintaining environmental stability, from mitigating greenhouse gas (GHG) emissions [9] to soil fertility [10] and wastewater treatment [11]. By leveraging genome-resolved metagenomics, researchers can harness microbial potential for sustainable agriculture, pollution control, and ecosystem restoration. Understanding these microbial functions is essential for developing strategies that promote biodiversity conservation and the long-term stability of natural and engineered ecosystems.

In this review, we will explore the impact of MAGs on microbial ecology and biogeochemical cycle research, emphasizing their role in carbon, nitrogen, and sulfur metabolism with a particular focus on their implications for environmental sustainability. We will discuss methodological advances in genome-resolved metagenomics, highlighting commonly used sequencing strategies, assembly, and binning approaches. Finally, we will address several key limitations, including assembly biases and incomplete metabolic reconstructions, and propose strategies to enhance the accuracy and applicability of MAG-derived insights.

### 1.1. Definition and Significance of MAGs

**Figure 1:**
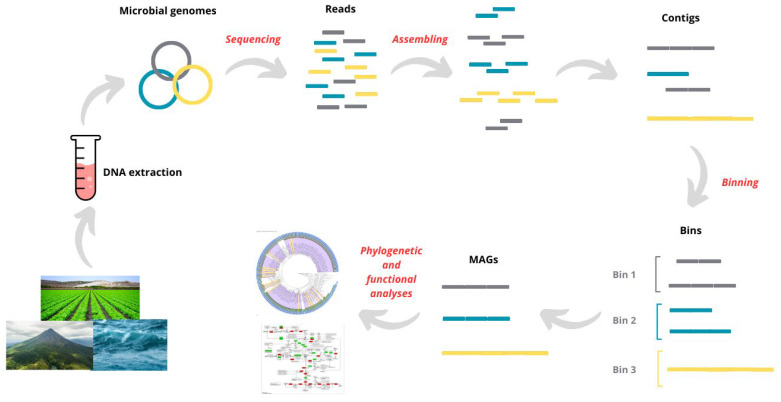
Workflow for MAG recovery. Figure illustrates the bioinformatic pipeline used to reconstruct MAGs from environmental samples. DNA is extracted from diverse ecosystems, including agricultural soils, marine environments, and volcanic regions. Sequencing generates short reads, which are subsequently assembled into contigs. Binning algorithms cluster contigs into discrete genome bins, representing draft microbial genomes. Recovered MAGs undergo phylogenetic and functional analyses to infer taxonomic affiliations and metabolic potential. This approach enables characterization of uncultivated microbial populations and their roles in biogeochemical cycles. Key steps in process (sequencing, assembling, binning, and analyses) are highlighted in red.

MAGs can be considered complete or near-complete microbial genomes reconstructed entirely from complex microbial communities. The DNA sequences obtained from an environmental sample are first assembled into longer contiguous sequences, known as contigs, which are then classified through a binning process, grouping them into bins that represent individual genomes (Figure 1). Unlike traditional methods, which only yielded genomes from cultivable microorganisms, MAGs allow the recovery of genomes from entirely novel or rare taxa, also known as “microbial dark matter” [12], without the need for laboratory cultivation, thereby enriching the Tree of Life as we currently understand it. A recent study on the diversity of metagenomic sequences revealed that only a tiny fraction of the overall biodiversity account for cultivated taxa, 9.73% in bacteria and 6.55% in archaea, whereas MAGs represent 48.54% and 57.05%, respectively [13].

Moreover, MAGs facilitate the detection of biosynthetic gene clusters (BGCs), which are co-localized sets of genes responsible for the production of specialized metabolites such as antibiotics, siderophores, and quorum-sensing molecules. These compounds are ecologically relevant, mediating microbial interactions, defense, and communication, and their study offers numerous advantages for studying microbial metabolism and diversity. It enables the direct linkage of specific metabolic functions to individual microorganisms—an achievement that was exceedingly difficult just a few years ago. Consequently, MAGs provide a deeper understanding of biogeochemical cycles and microbial metabolism at large. Additionally, MAGs allow the exploration of microbial relationships and their functional roles within ecosystems, offering a more comprehensive understanding of microbial contributions to environmental processes.

### 1.2. Historical Context: Transition from Marker Gene Surveys to Whole-Genome Recovery

The study of microbial communities has evolved significantly in recent years, transitioning from the predominant use of genetic markers, such as 16S rRNA, *rpoB*, and *recA*, to the emergence of metagenomics. In the early years of molecular ecology, the most widely used molecular marker was the 16S rRNA gene coupled with several molecular methods (i.e., DGGE, Denaturing Gradient Gel Electrophoresis; RFLP, Restriction Fragment Length Polymorphism; RAPD, Random Amplified Polymorphic DNA; RT-PCR, Real-Time Polymerase Chain Reaction) [14]. This gene presents a key advantage as it is universally present in all microorganisms within the domains Archaea and Bacteria, both of which constitute essential components of microbial communities. Additionally, the 16S rRNA gene contains both highly conserved and variable regions, allowing for in-depth sequence analysis with sufficient phylogenetic resolution to classify and identify microorganisms [15].

By directly amplifying this gene through PCR from an environmental sample, the characterization of the microbial community members became possible without requiring cultivation. This major breakthrough provided access for the first time to the uncultivable microbial diversity present in a given sample. However, despite this significant advancement, the technique had a key limitation: it could not provide insights into the potential functional roles of microorganisms, as it relied solely on a single ribosomal gene sequence rather than a complete genome. In addition, the use of only the 16S rRNA sequence gave rise to different concerns such as the lack of phylogenetic resolution to resolve the deepest nodes [16,17], the presence of multiple heterogeneous copies of the gene within a given genome [18,19,20], and the formation of chimeric PCR amplification products from complex environmental samples [21]. Therefore, to overcome these issues, it is advisable to use additional gene markers [22].

The advent of high-throughput sequencing in the early 2000s marked a radical shift in molecular ecology studies. Rather than relying solely on a handful of genetic markers, it became possible to sequence thousands of genes, granting access to the metagenome, the collective hereditary material present in an environmental sample [23]. This approach, known as shotgun metagenomics, enabled the inference of numerous microbial functions, as well as the characterization of community diversity. Moreover, it shed light on the metabolic potential of microbial communities, facilitating the discovery of novel genes and metabolic pathways. Shotgun metagenomics laid the foundation for the concept of MAGs. The first study to apply this concept was conducted by Tyson et al. in an acid mine drainage environment of the Richmond mine at Iron Mountain, California (USA) where the near-complete genomes of the archaeon *Ferroplasma* and the bacterium *Leptospirillum* were successfully reconstructed [24]. This study also allowed the inference of their symbiotic interactions and metabolic pathways within biofilms.

## 2. Methods for Recovering and Analyzing MAGs

### 2.1. Sample Selection and DNA Extraction Considerations

Sampling is the first step in any MAG research (Figure 1) and sample selection should be tailored to the objectives of the study, whether it is aimed at discovering novel taxa, identifying new BGCs, or characterizing the specific functions of a microbiome for ecological research. Appropriate sampling and storage protocols are crucial for preserving microbial community structure and nucleic acid integrity. For example, in host-associated microbiomes, especially gut content from animals, it is essential to collect samples using sterile tools and to place them in sterile, DNA-free containers. Samples should be stored at −80 °C as soon as possible or, alternatively, stabilized using nucleic acid preservation buffers (e.g., RNAlater or OMNIgene.GUT) when freezing is not feasible. Avoiding repeated freeze–thaw cycles is critical, as these can cause DNA shearing and impact downstream assembly quality. Additionally, standardized protocols for fecal or gut content sampling, including time of collection relative to feeding and host handling, can minimize biological variability. Improper handling at this stage can compromise community profiles, reduce genome completeness, and limit the functional interpretation of MAGs. Other key factors to consider include the following:

- Microbial diversity and biomass: Some environments, such as soils or marine sediments, may exhibit high microbial diversity and require deep sequencing to identify rare taxa. Conversely, other environments, such as extreme habitats or bioreactors, may have lower diversity and could benefit from culture enrichment or selective filtration strategies.
- Microbial activity and functional potential: Temporal sampling strategies, microcosm experiments, or stable isotope probing (SIP) can provide valuable insights into active microbial populations at a given time and their associated functions.
- DNA yield and quality: For genome assembly and binning, it is preferable to use high-molecular-weight DNA. This requires extraction protocols that minimize DNA fragmentation and degradation while also reducing contamination from host DNA, which is particularly critical for gut or host-associated samples.

### 2.2. Sequencing Technology Selection and Its Impact on MAG Quality

Another critical factor to consider is the choice of sequencing technology, as it significantly influences the quality of genome assembly and the recovery of high-quality MAGs. Sequencing technologies can be broadly categorized into short-read sequencing and long-read sequencing, each with its own advantages and limitations, as detailed in Table 1.

#### 2.2.1. Short-Read Sequencing Technologies

Short-read sequencing platforms, such as Illumina and BGI-Seq, generate reads ranging from 100 to 300 bp in length [25]. Their high accuracy and cost-effectiveness make them the preferred choice for large-scale studies. However, the relatively short read length poses challenges when analyzing complex microbial communities or highly repetitive DNA regions, as it can lead to incomplete or highly fragmented MAGs.

#### 2.2.2. Long-Read Sequencing Technologies

Long-read sequencing platforms, such as Pacific Biosciences (PacBio) and Oxford Nanopore Technologies (ONT), produce long DNA fragments spanning several kilobases up to 10–12 kb of mean read length [25,26]. This capability is particularly beneficial for sequencing repetitive regions or complex genomic architectures, leading to the recovery of more complete MAGs. However, it is important to note that long-read platforms generally have a higher per-read error rate compared to short-read technologies. To mitigate this limitation, increasingly sophisticated bioinformatics algorithms and correction tools are being developed to improve sequencing accuracy [27].

#### 2.2.3. Hybrid Approaches

A powerful strategy to leverage the advantages of both sequencing technologies is hybrid sequencing, which combines short- and long-read platforms. This approach enhances both accuracy and genome completeness, resulting in higher-quality MAGs [28] and has shown to significantly improve the resolution of complex microbial communities in environmental samples, making them an increasingly popular choice in metagenomics studies [29].

#### 2.2.4. Other Approaches

In recent years, other alternative culture-independent strategies have been developed:

- Hi-C and Proximity: This method preserves the three-dimensional organization of DNA within microbial cells and relies on crosslinking DNA fragments that are in close spatial proximity within intact cells, followed by restricting enzyme digestion and sequencing of ligated fragments [30].
- Single-cell metagenomics (SCM): This strategy enables the genomic characterization of individual microbial cells without the need for assembly based binning. This approach involves fluorescence-activated cell sorting (FACS) or microfluidics-based isolation of single cells, followed by whole-genome amplification (WGA) and sequencing [31].

### 2.3. De Novo Assembly

Reconstructing complete individual genomes from the DNA of complex microbial communities is a highly challenging task. As such, de novo assembly represents a crucial step in metagenomic workflows, enabling the reconstruction of genomes without the need for a reference genome (Figure 1). To achieve this goal, advanced computational assemblers such as MEGAHIT [32] and metaSPAdes [33] have been specifically designed to efficiently piece together sequencing reads. MEGAHIT is an ultrafast and memory-efficient assembler that uses a succinct de Bruijn graph representation, allowing it to handle the massive datasets typically generated in metagenomics studies [32]. Unlike other assemblers, MEGAHIT processes all sequencing data collectively, eliminating the need for preprocessing steps such as partitioning or normalization. On the other hand, SPAdes was initially developed for single-cell sequencing projects [34]. However, when applied to complex microbial communities, this tool exhibited high memory consumption, making it impractical for large-scale environmental metagenomics. To address this challenge, the same development team introduced metaSPAdes in 2017, an assembler specifically optimized for metagenomic datasets [34]. metaSPAdes incorporates advanced computational strategies designed to accurately assemble polymorphic fragments from highly diverse samples. This approach significantly improves the reconstruction of high-quality MAGs across various environmental and host-associated microbiomes.

### 2.4. Computational Binning Strategies

The reconstruction of metagenomes derived from complex microbial communities is performed through a process known as binning, which involves grouping assembled contigs into bins that correspond to individual genomes (Figure 1). To enhance the efficiency of this process, various computational tools have been developed, each one employing specialized algorithms for optimization of genome reconstruction.

#### 2.4.1. MetaBAT

MetaBAT (Metagenome Binning with Abundance and Tetranucleotide Frequencies) is an automated tool designed for the efficient binning of metagenomic contigs [35]. It calculates probabilistic abundance distances based on tetranucleotide frequency (TNF) patterns. Using these parameters, MetaBAT groups contigs into bins, which are likely to represent individual genomes. This tool is highly scalable, capable of handling large-scale datasets with millions of contigs. Its updated version, MetaBAT2, introduces an improved binning algorithm that enhances efficiency by eliminating the need for manual parameter adjustment.

#### 2.4.2. CONCOCT

This bioinformatics tool is an unsupervised binning algorithm that clusters metagenomic contigs based on k-mer frequencies and coverage across multiple samples [36]. It employs a Gaussian Mixture Model to classify contigs, with an additional script enabling evaluation based on single-copy genes. This approach allows for the effective separation of contigs from closely related species, making it particularly useful for studying highly diverse microbial communities, such as soils. However, its performance can be influenced by data quality and the intrinsic characteristics of the studied community.

#### 2.4.3. MaxBin

Another widely used binning tool is MaxBin, which distinguishes contigs into different bins based on coverage levels and tetranucleotide frequency profiles [37]. The updated MaxBin 2.0 version incorporates additional features to improve the recovery of genomes from co-assembled metagenomic datasets derived from multiple samples.

Each of these binning algorithms presents distinct advantages, and their effectiveness depends on dataset complexity, community structure, and sequencing depth. Given these factors, it is often recommended to integrate multiple binning approaches in order to refine bins to achieve higher completeness and less contamination, the two primary parameters used to assess the quality of MAGs. The preferred tool for this purpose is DAS Tool, an automated method that combines binning outputs from multiple algorithms (e.g., CONCOCT, MaxBin2, MetaBAT), using a de-replication, aggregation, and scoring strategy to generate an optimal, non-redundant set of bins from a single metagenomic assembly [38].

### 2.5. Validation and Taxonomic Classification of MAGs

In metagenomic studies, the validation and refinement of MAGs are critical steps to ensure that the reconstructed genomes are complete and free from contamination by sequences from other genomes. Additionally, this phase is essential for achieving an accurate taxonomic classification of MAGs.

**Figure 2:**
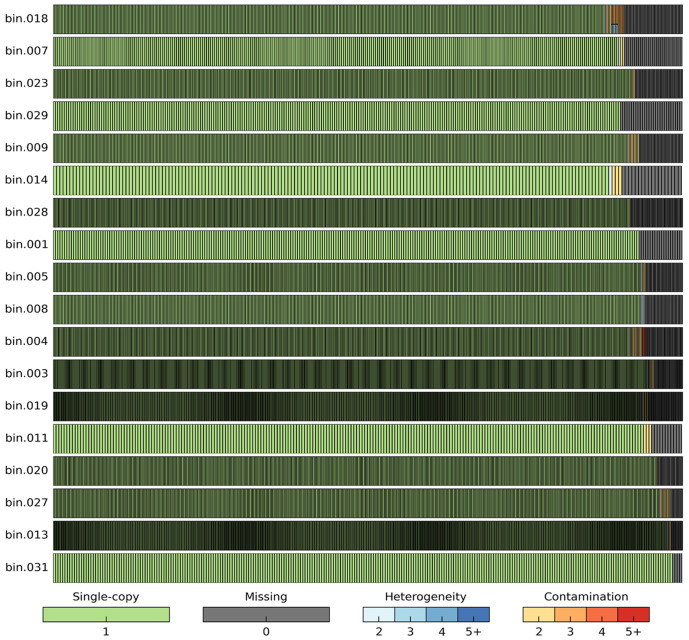
Quality assessment of MAGs using CheckM [39]. Each row represents individual MAG, with vertical bars corresponding to presence and status of single-copy marker genes. Green bars indicate single-copy genes, while dark gray bars represent missing genes. Contamination, inferred from presence of multiple copies of marker genes, is denoted by gradient from yellow to red. Heterogeneity, reflecting sequence variation within marker genes, is shown in blue. Bottom of color scale represents degree of heterogeneity and contamination, ranging from low (light) to high (dark).

To assess the quality of MAGs obtained from metagenomic data, a widely used computational tool is CheckM, which estimates both genome completeness and contamination levels [39]. This tool relies on a set of ubiquitous, single-copy marker genes that are conserved within a given phylogenetic lineage. By analyzing both the presence and redundancy of these marker genes, CheckM provides quality metrics that facilitate the selection of MAGs for downstream analyses (Figure 2).

For MAG filtering, several standard thresholds of completeness and contamination have been proposed. A widely accepted classification, outlined by Bowers et al. [40], defines MAG quality as follows:

- High-quality MAGs: >90% completeness, <5% contamination.
- Medium-quality MAGs: >50% completeness, <10% contamination.
- Low-quality MAGs: <50% completeness, <10% contamination.

These quality thresholds provide a systematic approach for evaluating MAGs and ensuring their suitability for further genomic and ecological studies.

For taxonomic identification, GTDB-Tk is a computational tool designed to classify bacterial and archaeal genomes using the Genome Taxonomy Database (GTDB), which provides objective taxonomic assignments and can efficiently process thousands of genomes and MAGs in parallel [41]. In fact, GTDB-Tk has shown high consistency when compared to manual classifications and it is considered a key tool in microbial research, as it facilitates the analysis of genetic diversity in environmental samples. Furthermore, it is particularly valuable for integrating MAGs into a broader taxonomic framework, allowing comparative analyses and ecological studies.

## 3. The Role of MAGs in Biodiversity Conservation and Sustainability

As previously discussed, microbial taxonomy studies have traditionally relied on culture-based methods and genetic markers, such as 16S rRNA gene sequencing. While these approaches have been informative, they are inherently limited in capturing the vast diversity of uncultivable microbial lineages and their functions. These methodological constraints have resulted in significant gaps in our understanding of microbial phylogeny, particularly in extreme environments where cultivation is often ineffective [7]. In fact, many of these microorganisms thrive in extreme environments, some of which are particularly fragile, including deep-sea hydrothermal vents [42], glacial ecosystems [43], and subsurface sediments [44], where they play a fundamental role in biogeochemical cycles and ecosystem resilience. In this context, MAGs have successfully circumvented these limitations, enabling the reconstruction of individual genomes from metagenomic data and thereby expanding our current view of life’s diversity.

A striking example of this paradigm shift is the discovery of the Candidate Phyla Radiation (CPR). This group was first identified through large-scale metagenomic analyses [45] and represents a deeply branching lineage distinct from previously characterized bacterial phyla. CPR bacteria are distinguished by their small genomes, minimal metabolic capabilities, and potentially symbiotic lifestyle, which likely contributed to their elusiveness using traditional techniques. This newly recognized radiation challenges our current understanding of bacterial diversity and evolutionary trajectories, suggesting that reductive evolution and host dependency are far more widespread among prokaryotes than previously assumed.

Similarly, the discovery of the Asgard archaea through genome-resolved metagenomics [46] has provided unprecedented knowledge of the origins of eukaryotes. These archaea, which include lineages such as *Lokiarchaeota*, *Thorarchaeota*, and *Odinarchaeota*, exhibit genetic features bridging the gap between prokaryotes and eukaryotes. Specifically, they encode homologues to eukaryotic coat proteins involved in vesicle biogenesis and to components of the membrane trafficking systems, supporting the hypothesis that eukaryotes evolved from an archaeal ancestor through an endosymbiotic event with an ancestral alphaproteobacterial (mitochondrial) cell [46]. The discovery of these archaea has reshaped the three-domain model of life, providing a genomic framework for reconstructing the evolutionary transitions that led to eukaryogenesis.

Expanding the Tree of Life through MAG-based studies not only enhances our understanding of evolutionary processes but also informs conservation strategies aimed at preserving microbial diversity as a fundamental component of ecological stability. Properly characterizing these microbial lineages provides insights into the adaptive strategies that enable life to persist under extreme conditions—knowledge that is becoming increasingly relevant in the face of climate change and habitat degradation. Integrating MAGs into conservation biology and sustainability research holds significant potential for biotechnology and ecosystem restoration. Many newly identified microbial taxa encode unique enzymes and metabolic pathways, which could drive innovations in renewable energy, bioremediation, and sustainable agriculture. A clear example in industrial applications is the discovery of thermostable enzymes from extremophilic microbes adapted to high temperatures and acidic environments [47]. Harnessing the genetic and metabolic potential of microbial communities could lead to novel strategies to address environmental challenges through bio-based solutions.

## 4. Functional Insights: Biogeochemical Cycles and Ecosystem Sustainability

Microorganisms are the primary drivers of Earth’s biogeochemical cycles, mediating the transformation of key elements such as carbon, nitrogen, and sulfur [48,49]. Through MAGs, metagenomics has led to unprecedented advancements in our understanding of these biological processes, enabling the identification of novel microbial lineages and metabolic pathways that underpin major biogeochemical cycles [50,51]. Given the accelerating impact of anthropogenic activities on ecosystem stability and climate, elucidating the microbial contributions to these cycles is critical for sustainable environmental management. Thus, MAG-based studies may provide essential understanding of the microbial functions that regulate greenhouse gas emissions, carbon sequestration, and soil fertility. These findings have significant implications for climate change mitigation and sustainable agriculture.

In addition to their critical roles in elemental cycling, the genetic and metabolic potential of microbial communities offers promising opportunities for applied environmental and agricultural biotechnology. Therefore, recent MAG-based studies have revealed changes in microbial gene content, suggesting potential for developing feed additives that modulate gut microbiota [52,53]. These approaches aim to enhance animal health and productivity via microbiological prevention, reducing the need for antibiotics and thereby limiting the spread of multidrug resistance (MDR) genes in natural ecosystems.

### 4.1. Microbial Contributions to Carbon Cycle

Microbial communities play a fundamental role in regulating the carbon cycle by mediating processes such as carbon fixation and organic matter degradation, which directly influence the emission or sequestration of greenhouse gases like CO_2_ and CH_4_. MAG-based studies have unveiled novel microbial taxa involved in methane oxidation and carbon sequestration in the deep sea, two fundamental processes in regulating CO_2_ and CH_4_ levels.

One of the most notable discoveries in this field, achieved through a metagenomics-driven approach, was the identification of *Candidatus* Methylomirabilis, an anaerobic methane-oxidizing bacterium that paradoxically generates intracellular oxygen from nitrite reduction, enabling methane oxidation via a pathway typically associated with aerobic methanotrophs [54]. This metabolic adaptation is particularly relevant in methane-emitting environments, such as wetlands, rice paddies, and other anoxic habitats. By reducing CH_4_ emissions into the atmosphere, these microbes could contribute to greenhouse gas mitigation, a key aspect of environmental sustainability. A recent study to obtain MAGs related to the carbon cycle recovered 17 MAGs from microbial communities derived from amazonian soils showing carbohydrate-active enzyme genes (CAZymes) and others associated with the biogeochemical cycles of nitrogen, sulfur, and methane [55]. Another study identified 57 MAGs representing putative methanogens, methanotrophs, and methylotrophs involved in methane and C1 compound cycling out of 1233 recovered genomes from subsurface floodplain sediments [44]. A recent study employing a hybrid sequencing approach combining Illumina and PacBio technologies revealed that prokaryotes play a central role in the carbon cycle within mangrove forests [56]. More specifically, several MAGs associated with three distinct carbon fixation pathways were recovered, including the Calvin–Benson–Bassham (CBB) cycle, the reverse tricarboxylic acid (rTCA) cycle, and the Wood–Ljungdahl (WL) pathway, with dominant representatives from bacteria, archaea, and fungi [56]. These ecosystems represent transitional environments between terrestrial and aquatic ones, functioning as intertidal coastal systems with a significant influence on climate change dynamics and environmental sustainability.

In marine ecosystems, deep-sea sediments can serve as long-term carbon sinks, although the mechanisms involved in carbon sequestration remain poorly understood. MAG-based analyses have the potential to identify novel heterotrophic and chemoautotrophic lineages involved in organic matter degradation and carbon fixation in deep-sea environments, where microbial communities are pivotal in shaping global biogeochemical cycles [57].

For instance, microbial communities contribute to deep-ocean carbon storage by facilitating the biological carbon pump, a process that transfers organic carbon from the surface to deep-sea sediments through particulate organic matter sinking and microbial remineralization [58], a key factor in buffering the rise in atmospheric CO_2_ levels. MAG-based analyses have uncovered extensive taxonomic and functional diversification within the abundant marine *Roseobacter* RCA cluster. This group plays a significant role in the marine carbon cycle, and its study provides valuable insights into biogeochemical processes in the oceans [59]. By expanding our understanding of the role of microbes in carbon fluxes, MAG-based research may propose alternative strategies to enhance carbon sequestration via microbial biotechnology applications.

### 4.2. Microbial Contributions to Nitrogen Cycle

For ecosystem sustainability, the nitrogen cycle represents a fundamental process, as it regulates nitrogen availability for primary producers. However, anthropogenic activities, particularly the excessive use of fertilizers, can severely disrupt this cycle, leading to eutrophication and increased greenhouse gas emissions. In aquatic environments, ammonia-oxidizing archaea (AOA) are key players in the conversion of ammonia into oxidized forms of nitrogen. Metagenomic studies have revealed the presence of high-quality AOA MAGs such as *Nitrosopumilus* and *Nitrosomarinus*-like lineages, which may play a key role in the global nitrogen cycle [60].

Among the most significant discoveries in this field is the identification of a crenarchaeote capable of performing chemolithoautotrophic growth by aerobically oxidizing ammonia to nitrite, confirming that nitrifying marine archaea are essential to nitrogen cycling within marine ecosystems [61]. Unlike ammonia-oxidizing bacteria (AOB), which require higher ammonia concentrations, AOA thrive in oligotrophic environments and have adapted to various ecological niches, including strongly acidic soils where specific lineages, such as *Nitrosotalea devaniterrae*, play a role in nitrification [62]. Their widespread distribution suggests that AOA are crucial in nitrogen turnover across oceans, agricultural soils, and aquatic systems [63]. Given the critical role of nitrification in soil nitrogen dynamics, understanding the ecological functions of these archaea is essential for developing more sustainable fertilization strategies that optimize nitrogen retention while minimizing greenhouse gas emissions (e.g., nitrous oxide, N_2_O).

Furthermore, MAG-based studies have identified previously uncharacterized denitrifying bacteria and archaea involved in nitrogen removal, particularly in wastewater treatment systems and wetland ecosystems. For example, certain MAG analyses identified bacteria in activated sludge harboring abundant genes associated with the denitrification pathway [64]. Another study recovered over 1000 high-quality MAGs from wastewater treatment plants using a hybrid sequencing strategy, which included denitrifiers primarily affiliated with *Gammaproteobacteria* [65]. Enhancing nitrogen removal efficiency through bioremediation strategies that leverage these microbial communities could contribute to reducing the environmental footprint of nitrogen pollution generated by industrial and agricultural activities.

### 4.3. Microbial Contributions to Sulfur Cycle

Sulfur metabolism is a key component of both marine and terrestrial ecosystems, closely linked to carbon and nitrogen cycles, as it influences soil fertility, ocean chemistry, and atmospheric sulfur dynamics. Consequently, sulfur-reducing and sulfur-oxidizing microorganisms have a significant impact on these processes. Metagenomic studies in several environments have identified 13 bacterial and archaeal phyla with the capacity for sulfate/sulfite reduction in their genomes, from which 8 were candidate phyla lacking cultured representatives [66]. Deep-sea hydrothermal vents are hotspots for sulfur cycling, where chemolithotrophic microorganisms obtain energy from oxidation of reduced sulfur compounds, fueling primary production in these extreme environments. The reconstruction of 58 MAGs derived from tropical and subtropical deep oceans revealed unique non-cyanobacterial diazotrophic bacteria as well as chemolithoautotrophic prokaryotes involved in potentially relevant biogeochemical processes including sulfur oxidation [67]. Moreover, these microorganisms contribute to deep-sea primary production through chemosynthesis, sustaining ecosystems that thrive in the absence of sunlight. Their metabolic pathways significantly influence global sulfur fluxes and oceanic nutrient cycles, with profound implications for marine biodiversity and biogeochemical equilibrium.

Meanwhile, in terrestrial environments, MAG-based studies have revealed the presence and functional potential of sulfur-metabolizing microorganisms in a glacial ecosystem [43], a permafrost core from Svalbard [68], terrestrial hot springs [29,69], and ancient Andean lake sediments [70]. These discoveries highlight the potential of sulfur-metabolizing microorganisms for the development of microbial-based soil amendments, which could enhance soil fertility and agricultural sustainability while reducing the environmental impact of intensive farming practices.

## 5. Challenges and Solutions in MAG Recovery

MAGs are inherently subject to errors that arise during DNA extraction, sequencing, and assembly processes. These biases can lead to contamination and the loss of certain genomic fragments. One of the most significant challenges is uneven sequencing depth, which disproportionately favors high-abundance taxa at the expense of rarer or lower biomass taxa due to their lower representation in metagenomic datasets [71]. Additionally, a major issue stems from assembly algorithms struggling with highly similar genomic sequences, showing more performance at high taxonomic ranks and less precision below family level [72]. In recent years, long-read sequencing technologies such as PacBio have significantly improved the resolution of complex microbial communities, enabling the generation of longer contigs with fewer gaps and improved assembly accuracy [73]. Furthermore, hybrid assembly strategies that integrate short- and long-read data such as the Illumina HiSeq-PacBio hybrid metagenomic approach have demonstrated improvements in MAG contiguity, completeness, and strain-level resolution [28,29]. Specific hybrid assemblers such as SPAdes-Hybrid [33], MaSuRCA [74], and OPERA-MS [75] have been successfully applied to resolve complex metagenomes by leveraging both sequencing modalities.

Another major limitation of MAG-based studies is the challenge of accurate taxonomic assignment for reconstructed genomes. Reference databases such as GTDB while comprehensive, remain incomplete, particularly for novel or uncultured microbial lineages. As a result, many MAGs remain unclassified beyond the phylum or class level, potentially constraining ecological interpretations. However, the increasing availability of curated reference genomes and the continuous refinement of phylogenomic frameworks such as GTDB have significantly improved taxonomic resolution [76]. Advances in taxonomic classification tools, including GTDB-Tk [41] and phylogenetic placement methods like PhyloPhlAn [77], are helping to integrate novel MAGs into existing microbial phylogenies with greater accuracy. Nevertheless, ongoing efforts to expand reference datasets and validate taxonomic assignments through single-cell genomics and cultivation-based approaches remain instrumental for reducing classification ambiguities in metagenomic studies.

Due to the inherent limitations of MAGs, metabolic pathway annotations often remain incomplete. The fragmented nature of environmental DNA, sequencing errors, and assembly challenges can lead to the partial or missing recovery of key metabolic genes, ultimately constraining accurate microbial metabolic reconstructions [78]. Additionally, biases in sequencing depth and genome binning may disproportionately affect the representation of certain functional pathways, leading to gaps in biogeochemical cycle analyses. To mitigate these challenges, genome binning algorithms that we reviewed here such as MetaBat2, CONCOCT, MaxBin2, and DAS Tool have been developed to enhance genome completeness while minimizing contamination. However, even high-quality MAGs may lack key genes due to the inherent difficulties in assembling repetitive or low-coverage genomic regions. Integrating multi-omics approaches—including metatranscriptomics, metaproteomics, and metabolomics—provides a robust strategy for validating metabolic predictions and linking genomic potential to actual microbial activity [79]. Furthermore, metabolic reconstruction tools and databases such as KEGG [80] and DRAM [81] facilitate pathway completion by leveraging functional annotations from reference genomes.

## 6. Concluding Remarks

MAGs have transformed microbial ecology by enabling genome-resolved studies of uncultured microorganisms across diverse ecosystems. These advances have not only expanded known microbial diversity but also revealed previously unrecognized functional traits, deepening our understanding of microbial contributions to biogeochemical cycles. The ability to reconstruct complete and near-complete genomes directly from environmental samples has provided a window into the metabolic potential of key microbial players in carbon, nitrogen, and sulfur cycling.

From a sustainability perspective, these discoveries are critical. MAGs have identified microorganisms that drive methane oxidation, carbon sequestration in marine sediments, and nitrogen transformations in soil and aquatic ecosystems. Additionally, sulfur-metabolizing microorganisms identified through MAG-based studies play essential roles in sulfur oxidation and reduction, particularly in deep-sea hydrothermal vents and terrestrial soils. These microbial processes influence atmospheric sulfur fluxes, soil fertility, and oceanic nutrient cycles, underscoring their ecological significance. Collectively, these findings not only refine our understanding of elemental fluxes but also have direct applications in climate change mitigation, sustainable agriculture, and bioremediation strategies. The use of MAGs to characterize microbial communities in wastewater treatment plants, degraded soils, and extreme environments highlights their role in applied environmental management.

Despite these advances, methodological challenges remain. Assembly biases, contamination in genome bins, and incomplete metabolic reconstructions continue to limit the ecological interpretation of MAGs. Addressing these limitations requires further advancements in short- and long-read sequencing, assembly, and binning algorithms. Expanding reference genome databases and integrating MAGs with other omics approaches, such as metabolomics and metatranscriptomics, will further enhance their utility in microbial ecology and biotechnology.

The continued refinement of genome-resolved metagenomics will be essential for linking microbial diversity to ecosystem function and stability. As environmental pressures intensify, leveraging MAG-based insights will provide essential tools for biodiversity conservation and the development of sustainable biogeochemical interventions.

## Figures and Tables

**Table 1:** Metagenomic sequencing strategies for MAG recovery.

Sequencing Approach	Technology Examples	Advantages	Limitations
Short-Read Sequencing	Illumina (NovaSeq, HiSeq), BGI-Seq	- High accuracy, low error rates - Cost-effective for large-scale studies - Suitable for taxonomic profiling and functional annotation	- Short reads (100–300 bp) result in fragmented assemblies - Challenges in resolving repetitive and complex genomic regions
Long-Read Sequencing	Oxford Nanopore (MinION, PromethION) PacBio HiFi	- Produces long reads (10–100 kb), improving genome continuity - Resolves structural variations and operon structures in biosynthetic gene clusters (BGCs) - Enables recovery of complete genomes	- Higher error rates (especially for Nanopore) - More expensive than short-read sequencing - Requires high-quality DNA input
Hybrid Sequencing	Combination of Illumina/BGI-seq + Nanopore/PacBio	- Balances accuracy and read length - Enhances genome completeness and scaffolding - Suitable for recovering novel taxa and complex microbial communities	- Higher cost due to dual sequencing platforms - Computationally demanding hybrid assembly pipelines
Hi-C and Proximity Ligation	Hi-C metagenomics MetaPhase	- Improves genome binning accuracy by linking genomic fragments from the same organism - Enhances MAG contiguity and taxonomic resolution	- Requires specialized library preparation - Still under development for metagenomic applications
Single-Cell Metagenomics	Fluorescence-Activated Cell Sorting (FACS) Microfluidics	- Enables genome reconstruction of rare and unculturable microbes - Complements MAGs by providing high-quality individual genomes	- Requires whole-genome amplification, which may introduce biases - Limited scalability for complex microbiomes

## Data Availability

No new data were created or analyzed in this study. Data sharing is not applicable to this article.
